# High‐fidelity detection of crop biomass quantitative trait loci from low‐cost imaging in the field

**DOI:** 10.1002/pld3.41

**Published:** 2018-02-22

**Authors:** Darshi Banan, Rachel E. Paul, Max J. Feldman, Mark W. Holmes, Hannah Schlake, Ivan Baxter, Hui Jiang, Andrew D.B. Leakey

**Affiliations:** ^1^ University of Illinois at Urbana‐Champaign Urbana IL USA; ^2^ Donald Danforth Plant Science Center St. Louis MO USA; ^3^ USDA‐ARS Donald Danforth Plant Science Center St. Louis MO USA

**Keywords:** crop production, hemispherical photographs, high‐throughput phenotyping, Leaf Area Index, setaria

## Abstract

Field‐based, rapid, and nondestructive techniques for assessing plant productivity are needed to accelerate the discovery of genotype‐to‐phenotype relationships in next‐generation biomass grass crops. The use of hemispherical imaging and light attenuation modeling was evaluated against destructive harvest measures with respect to their ability to accurately capture phenotypic and genotypic relationships in a field‐grown grass crop. Plant area index (PAI) estimated from below‐canopy hemispherical images, as well as a suite of thirteen traits assessed by manual destructive harvests, were measured in a *Setaria* recombinant inbred line mapping population segregating for aboveground productivity and architecture. A significant correlation was observed between PAI and biomass production across the population at maturity (*r* ^2^ = .60), as well as for select diverse genotypes sampled repeatedly over the growing season (*r* ^2^ = .79). Twenty‐seven quantitative trait loci (QTL) were detected for manually collected traits associated with biomass production. Of these, twenty‐one were found in four clusters of colocalized QTL. Analysis of image‐based estimates of PAI successfully identified all four QTL hot spots for biomass production. QTL for PAI had greater overlap with those detected for traits associated with biomass production than with those for plant architecture and biomass partitioning. Hemispherical imaging is an affordable and scalable method, which demonstrates how high‐throughput phenotyping can identify QTL related to biomass production of field trials in place of destructive harvests that are labor, time, and material intensive.

## 1 INTRODUCTION

Current rates of yield gain are unlikely to meet the projected demands of global population growth and development (Ray, Mueller, West, & Foley, 2013; Tilman, Balzer, Hill, & Befort, 2011). High‐throughput phenotyping (HTP) techniques rapidly evaluate plant performance and leverage advances in genotyping (Bennetzen et al., 2012; He et al., 2014; Poland & Rife, 2012), the development of mapping populations (Casa et al., 2008; Doust, Kellogg, Devos, & Bennetzen, 2009; Li & Brutnell, 2011), and the design and analysis of quantitative genetic experiments to ultimately develop a predictive understanding of genotype‐to‐phenotype relationships (Morrell, Buckler, & Ross‐Ibarra, 2011; Myles et al., 2009). This understanding enables an accelerated and more targeted approach to crop improvement (Araus & Cairns, 2014; Furbank & Tester, 2011). Biomass production generates both the calories that are partitioned toward food consumption and the raw feedstock used for carbon‐efficient biofuels (Hudiburg et al., 2016). Biomass productivity per unit ground area must be maximized to ensure profitability and avoid displacement or disturbance of natural ecosystems (Somerville & Long, 2015). However, biomass is a complex trait that is difficult to assess in the field and usually is measured by destructive harvest (White et al., 2012). We addressed this challenge by testing the ability of hemispherical imaging to identify genomic regions associated with aboveground biomass production. The successful application of hemispherical imaging was evaluated with respect to its strong phenotypic correlation and high degree of QTL colocalization with directly validated destructive harvest traits.

Numerous remote sensing methods have been demonstrated to correlate with destructive measures of biomass production and can be considered proxy measurements (Casadesús & Villegas, 2014; Gitelson et al., 2003; Li et al., 2015; Parent et al., 2015). These include multispectral and hyperspectral indices of radiation reflected from crop canopies, as well as measures of canopy light distribution. Often, a combination of sensor outputs and additional processing techniques such as regression, inverse modeling, and multivariate analysis is required to produce relevant phenotypes (Fiorani & Schurr, 2013; White et al., 2012). Tanger et al. (2017) used a combination of tractor‐mounted multispectral reflectance and ultrasonic sensors to detect manually validated QTL associated with biomass in rice (Tanger et al., 2017). Similar measurements have been deployed in other field‐grown crops using ground vehicles, aerial vehicles, and gantries requiring investment in equipment that often exceeds $100,000s–$1,000,000s (Pauli et al., 2016; Vergara‐díaz, Zaman‐allah, Masuka, & Hornero, 2016). In addition to the expense of HTP equipment, many techniques under development require extensive research infrastructure, permits (e.g., flight authorization) and complex data analyses. Highly trained personnel are consequently needed to support both data acquisition and analysis. Unfortunately, these factors combine to mean that the majority of HTP techniques can only feasibly be used by large research institutions and companies. And, even in those organizations, deployment of HTP has to be limited to a few high‐priority projects. Cheap methods of HTP that rely on simpler technology could greatly increase how widely HTP is adopted, and support work in a broader diversity of environmental conditions and crops outside of the major growing regions of the world's staple crops.

**Figure 1:**
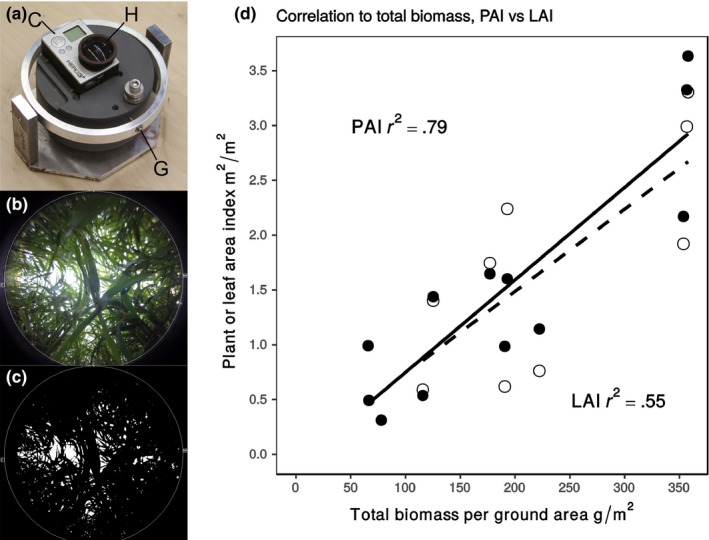
Customized hemispherical imaging system application to high‐throughput phenotyping of aboveground biomass production. (a), A hemispherical lens (H) fitted on a GoPro Hero3 +  digital camera (C) and mounted on a self‐leveling gimbal (G). The camera unit had maximum dimensions of 5.8×4×3.7 cm, and the full system was 11×15×13.3 cm. (b), The system was used to capture fully hemispherical images of a plant canopy. (c), Images were thresholded for analysis and estimation of Plant Area Index (PAI) using Delta‐T HemiView software. (d), These estimates were correlated with total biomass (filled symbols, solid line) and compared to that between Leaf Area Index estimated from destructive harvest and total biomass (open symbols, dashed line). Measurements made on parent lines A.10, B.100 and phenotypically intermediate RIL#161 together represent a diversity of growth habit and morphology seen across the population. Symbols correspond to single plots from which all images and measurements were collected 38, 44, 52, and 60 days after sowing. Correlation *r* ^2^ values are reported for both measurements

Hemispherical imaging captures the geometry of sky openings and models the attenuation of solar radiation by the canopy to estimate canopy properties, such as Leaf Area Index (LAI; leaf area per unit ground area) (Anderson, 1964; Rich, 1990). The ability to account for the influence of both stems and leaves allows its use for estimating Plant Area Index (PAI; aboveground plant tissue area per unit ground area) in herbaceous systems (Neumann, Den, & Shaw, 1989). Canopy properties estimated from hemispherical images use mechanistic and biophysical models rather than reliance on statistical relationships between sensor and subject. Therefore, they should be less context‐dependent and more widely applicable to different crops, growing conditions, and management practices than other methods that require a training model to relate remotely sensed data to traditional measures of crop productivity (e.g., Busemeyer et al., 2013). Traditionally, hemispherical photography equipment is tall, bulky, and not suited to crop HTP. In this study, a miniature remotely triggered digital camera designed for point‐of‐view action sport videography (GoPro Hero 3 + , GoPro Inc, San Mateo, CA, USA) was modified with a miniature hemispherical lens (1.39‐mm hemispherical lens, Quality Video Components LLC, Sparta, MI, USA) and mounted to a custom‐built self‐leveling gimbal (Figure 1a–c). The resulting system was small enough to fit between tight crop rows and below the crop canopy.

An F7 recombinant inbred line (RIL) mapping population with 186 genotypes generated from a cross between the cultivated *Setaria italica* and its weedy ancestor *Setaria viridis* provides an ideal platform for assessing the ability of hemispherical imaging to detect QTL related to aboveground biomass production due to its wide diversity of morphologies and multifold variation in biomass production (Bennetzen et al., 2012; Doust et al., 2009). As a model C_4_ grass emerging as a tool for systems‐level biology, the genus *Setaria* has the advantage of being closely related to C_4_ grass food and fuel crops such as maize, sorghum, miscanthus, and switchgrass, while having a smaller stature, faster life cycle, and diploid genome (Brutnell et al., 2010). Here, we use an inexpensive and simple, miniaturized system of hemispherical imaging and light attenuation modeling to identify the same set of key QTL for biomass production as traditional destructive harvest methods applied to a field‐grown *Setaria* mapping population. This provides a case study of a HTP technology that can deliver results for QTL mapping without high costs or complexity.

## 2 RESULTS AND DISCUSSION

First, a validation experiment determined whether the customized imaging system was suitable for HTP in *Setaria. Setaria viridis, Setaria italica,* and the phenotypically intermediate RIL #161 derived from crossing these two species were used as test material because they vary widely in canopy architecture and rate of biomass production. Each genotype was grown in eight replicated plots of which four were randomly chosen for measurement. An independent plot for each genotype was selected for collection of both hemispherical images and destructive canopy and biomass harvest measures on four dates distributed across the growing season to generate a wide range of canopy closures and biomasses with which to evaluate hemispherical imaging. Notably, total aboveground biomass correlated more strongly with PAI (*r* ^2^ = .79) than it did with destructively measured LAI (*r* ^2^ = .55; Figure 1d). This difference in correlation with total aboveground biomass likely results from the ability of hemispherical imaging to evaluate all visible plant elements versus destructive LAI accounting only for plant elements identified as leaf. This highlights the ability of hemispherical imaging to robustly assess the total amount of plant tissue over an area of ground across a diversity of short, herbaceous grass canopies in a nondestructive manner. Data are easy to acquire and analyze as the camera, lens, and analysis software are all commercially available.

Next, the ability of hemispherical imaging to detect QTL for biomass production was tested in a *Setaria* F7 RIL mapping population. 186 RILs were planted in an unreplicated randomized design with six check plots for each parent. Hemispherical images, manually measured morphological traits, and destructive harvest weight data were collected from the same plots of each genotype.

Results from manual measures of developmental, architecture, and biomass production traits showed that the segregating population was phenotypically diverse for a comprehensive set of destructive harvest traits assessed at maturity, including total aboveground biomass, tiller number, and height (Table S1).

**Figure 2:**
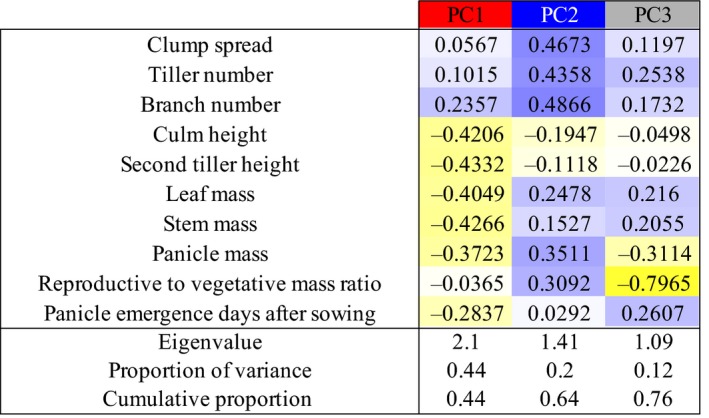
Principal component analysis of directly measured biomass traits. Trait loadings based on eigenvectors (color‐coded by direction and magnitude, blue: positive, yellow: negative) in each principal component (PC) appear to describe three orthogonal processes: biomass production (PC1), bushiness (PC2), and partitioning of biomass to vegetative versus reproductive structures (PC3). The individual and cumulative contributions of each PC are reported

Principal component analysis (PCA) was performed on directly measured traits—leaf mass, panicle mass, stem mass, branch number, tiller number, clump spread, culm height, tiller height, days after sowing until panicle emergence, and reproductive‐to‐vegetative mass ratio—to simplify the description of plant performance relative to hemispherical imaging estimates (Figure 2). The first three principal components (PCs) with eigenvalues greater than 1.00 together explain 76% of the variation in the dataset. The trait loadings based on eigenvectors in each of these three orthogonal PCs appear to describe three plant growth components: (i) biomass production, (ii) bushiness, and (iii) partitioning of biomass to vegetative versus reproductive structures. Days after sowing until panicle emergence varied significantly within the RIL population, loading moderately in both PC1 and PC3, but not strongly in any single PC. PC1 accounted for 44% of overall variation and approximates aboveground biomass production based on strong loadings for culm height, tiller height, leaf mass, panicle mass, and stem mass. PC2 accounted for 20% of variation and approximates plant bushiness based on strong loadings for clump spread, branch number, and tiller number. PC3 accounted for 12% of variation and approximates the partitioning of biomass to vegetative versus reproductive structures based on strong loadings for panicle mass and reproductive‐to‐vegetative mass ratio.

**Figure 3:**
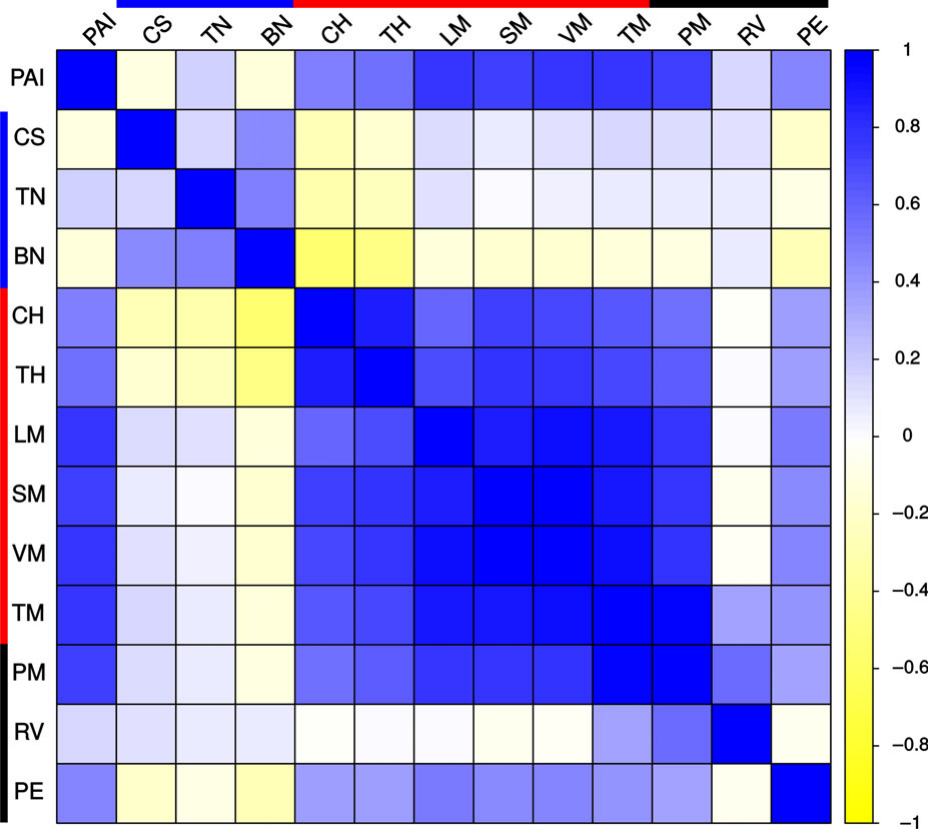
Correlation matrix of PAI and directly measured biomass traits. PAI correlates strongly and positively with traits associated with biomass production. Blue, yellow, and white cells represent positive, negative, and no correlation, respectively, between traits based on Pearson correlation coefficient values. Color bars at top and left indicate traits corresponding to bushiness (blue), biomass production (red), and partitioning of biomass to vegetative versus reproductive structures (black). PAI, plant area index; CS, clump spread; TN, tiller number, BN, branch number; CH, culm height; TH, second tiller height; LM, leaf mass per m^2^ ground; SM, stem mass per m^2^ ground; PM, panicle mass per m^2^ ground; VM, vegetative mass per m^2^ ground; TM, total mass per m^2^ ground; RV, reproductive‐to‐vegetative mass ratio; PE, panicle emergence days after sowing

These apparent descriptions, while not definitive, are biologically intuitive and provide a framework for interpreting the genetic and phenotypic attributes of a plant in the field. Correlation analysis of all traits measured shows that PAI correlated positively and strongly with total mass, vegetative mass, and with the other traits that loaded strongly into PC1 to describe biomass production (Figure 3).

**Figure 4:**
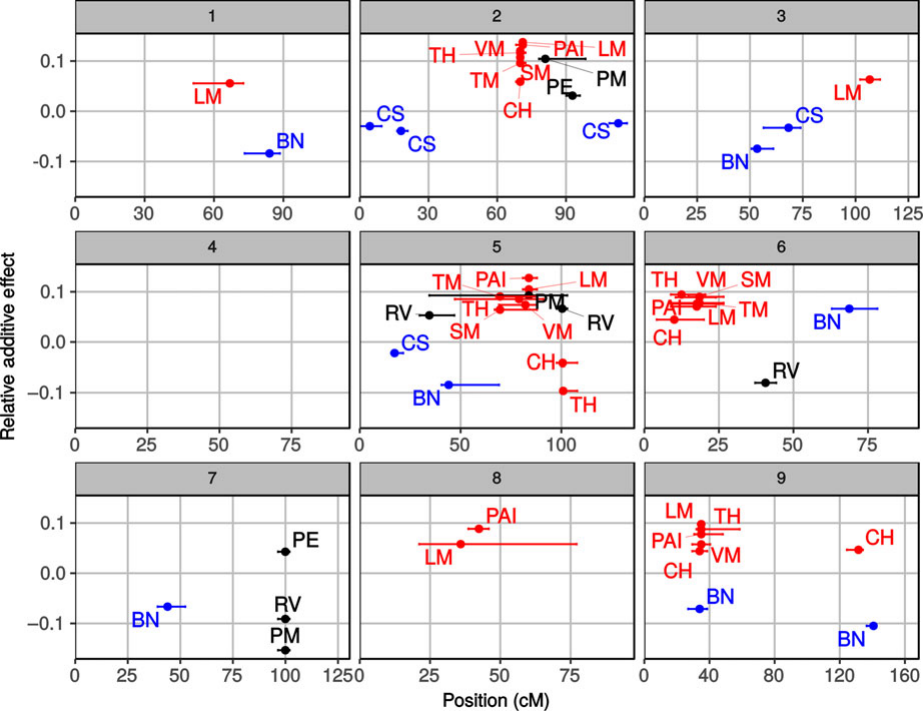
Quantitative trait loci (QTL) mapping for PAI and directly measured biomass traits. Panels 1–9 correspond to each chromosome in the *Setaria* genome. Additive effects relativized by phenotypic mean are plotted against centimorgan position for each QTL. Error bars represent the 1.5 LOD score confidence intervals for each QTL's location. Colored points represent QTL corresponding to biomass production (red) bushiness (blue) and partitioning of biomass to vegetative versus reproductive structures (black). PAI, plant area index; CS, clump spread; BN, branch number; CH, culm height; TH, second tiller height; LM, leaf mass per m^2^ ground; SM, stem mass per m^2^ ground; PM, panicle mass per m^2^ ground; VM, vegetative mass per m^2^ ground; TM, total mass per m^2^ ground; RV, reproductive‐to‐vegetative mass ratio; PE, panicle emergence days after sowing

Quantitative trait loci analysis detected 53 significant QTL across 12 traits (Table S2). The identified loci clustered in groups corresponding with the segregation of traits into the three different PCs (Figure 4). This suggests that the variation underlying the separation of PCs is driven by genetic programs related to biomass production, bushiness, and partitioning of biomass to vegetative versus reproductive structures rather than uncontrolled physiological or environmental factors.

Quantitative trait loci for PAI were colocated with all four of the hotspots of QTL for traits related to aboveground biomass production evaluated in destructive harvests. These four hot spots found on chromosomes 2, 5, 6, and 9 featured QTL for between five and seven traits, including PAI, total mass, vegetative mass, leaf mass, stem mass, culm height, and tiller height. All PAI and biomass productivity QTL within the four hotspots had positive additive effects. Together, these loci appear to represent the primary features of the genetic architecture of aboveground biomass production in *Setaria*. There was very little overlap between the location of these QTL hot spots for biomass production and QTL for traits associated with bushiness or vegetative‐to‐reproductive biomass ratio. Single QTL each for PAI and leaf mass colocalized on chromosome 8 and both had positive additive effects. Additionally, isolated QTL for leaf mass (chromosome 3 @ position 107 cM) and culm height (9@131) did not overlap with QTL for PAI. In contrast to all the other QTL for traits associated with biomass production, QTL for culm height and tiller height had negative additive effects and colocated with a single QTL for reproductive‐to‐vegetative mass ratio (5@100). There was also very close correspondence between QTL identified for PAI and other productivity traits in the study with an independent field experiment on the same RIL population in Oklahoma (Mauro‐Herrera & Doust, 2016). This provides strong evidence in support of using HTP of PAI to evaluate the genetic architecture of field‐grown grass crops biomass productivity.

Twelve QTL for traits related to plant bushiness and nine QTL for traits related to partitioning of biomass to vegetative versus reproductive structures were detected. Notably, there was minimal overlap between QTL for PAI and those for traits associated with plant bushiness and biomass partitioning rather than biomass production. So, while hemispherical imaging is a powerful tool for assessing the genetic architecture of productivity traits in the field, other more complex field imaging techniques or laboratory‐based methods will be needed to quickly phenotype plant architectural traits (Fernandez, Bao, Tang, & Schable, 2017; McCormick, Truong, & Mullet, 2016).

Quantitative trait loci for PAI captured the main features of the genetic architecture for directly measured traits related to biomass productivity (PC1), but in a nondestructive and far less laborious manner and without the need for intermediate calibration models. This was achieved with a minimal number of genotypes (186), replicates (1), and subsampling (2), confirming the method's power to detect the genetic components of variation in biomass productivity. In addition to its high‐detection fidelity, hemispherical imaging has the characteristics of an ideal, scalable HTP technique. Compared to the manual traits that it parallels, hemispherical imaging is very efficient (four person‐hours for image collection and seven for image analysis compared with 148 person‐hours for destructive biomass harvest and 15 person‐hours for sample weighing). The equipment used to collect hemispherical images is easy to operate, inexpensive, and commercially available. Image processing and analysis were accomplished using a commercially available software (HemiView, Delta‐T Devices Ltd, Cambridge, UK) on a standard desktop computer and did not require additional data streams. This simplicity, low‐cost, and universally applicable principle of operation means that the method could be deployed on diverse crops at any location by a small team of personnel with limited training. The equipment is compact and lightweight, meaning that it can also be deployed on a rover to further accelerate image collection, improve the signal‐to‐noise ratio, and uncover the temporal dynamics of biomass productivity.

## 3 MATERIALS AND METHODS

### 3.1 Validation experimental design

*Setaria viridis* and *Setaria italica,* the parents of the F7 RIL population, and the phenotypically intermediate RIL #161 were grown on the South Farms at the University of Illinois Urbana‐Champaign in summer 2014. The field site is rain‐fed, tile‐drained, has a deep, organically rich, Flanagan/Drummer series type soil. RIL #161 was selected as a phenotypic intermediate between *S viridis* and *S italica* because of its placement in the 50th percentile for both culm height and tiller number. The experiment was a randomized complete block design with all three genotypes replicated in eight plots of which four were randomly chosen for measurement. Each plot was 4 m^2^ with 25 cm grid spacing between plants. Data were collected from an independent plot of each genotype on four dates through the growing season. This resulted in significant variation in height, biomass production, canopy architecture, and PAI. Measured plots were not used for subsequent data collection.

First, nondestructive estimates of PAI were generated using HemiView software (Delta‐T Devices Ltd, Cambridge, UK) to analyze six canopy hemispherical photographs taken either within or between planting rows near the plot center under diffuse light conditions (predawn, dusk, or high cloud cover). Hemispherical photographs were taken with a GoPro Hero 3 +  digital camera (GoPro Inc, San Mateo, CA, USA) modified with a fully hemispherical lens (1.39‐mm hemispherical lens, Quality Video Components LLC, Sparta, MI, USA) and mounted on a custom‐built miniature self‐leveling gimbal. Second, 8 or 16 plants (depending on collection date) were harvested from each plot, and separated into leaf, stem, and reproductive tissues. Fresh leaves were laid flat and photographed with a digital SLR camera (Cannon EOS 7D, 50‐mm lens, Canon Inc, Huntington, NY, USA) alongside a scaling object to allow estimation of total leaf area using ImageJ (National Institutes of Health, Bethesda, MD, USA). All tissues were then dried at 65°C and weighed.

### 3.2 Quantitative trait loci experimental design

186 F7 recombinant inbred lines from an interspecific cross between *Setaria italica *× *Setaria viridis* were evaluated on the South Farms at the University of Illinois Urbana‐Champaign in summer 2014. Seeds were germinated in glasshouse and transplanted by hand into a mechanically tilled field 7 days after sowing. The experiment was an unreplicated randomized design with six check plots for each parent. Data were collected from a single plot (36 plants, 1 m^2^, 20 cm grid spacing between plants) of each RIL and six plots of each parent genotype.

### 3.3 Climate conditions

Over the duration of the validation experiment, the average air temperature was 21.74°C, the average humidity was 72.34%, and the cumulative rainfall was 37.23 cm. Over the duration of QTL experiment, the average air temperature was 19.37°C, the average humidity was 78.59%, and the cumulative rainfall was 18.85 cm.

### 3.4 Phenotyping

Panicle emergence was measured as the number of days after sowing at which the panicle head was seen past the collar of the flag leaf. The angle between the outermost tillers (i.e., clump spread) was measured in the field with a modified protractor 50 days after sowing. Nondestructive estimates of PAI were generated using HemiView software (Delta‐T Devices Ltd, Cambridge, UK) to analyze two canopy hemispherical photographs taken with a GoPro Hero 3 +  digital camera manually placed either within or between planting rows at the plot center under diffuse light conditions at dusk or dawn when the canopy was near maximum size prior to senescence at the end of the growing season, between 67 and 70 days after sowing. The GoPro camera was customized by replacing the standard lens with a 1.39 mm 190^°^ hemispherical lens and mounted on a custom‐built self‐leveling gimbal in order to insure the camera faced upward from horizontal. Image capture was triggered using a hand‐held Wi‐fi remote (GroPro Inc, San Mateo, CA, USA) such that the operator remained out of frame. The camera was consistently staged such that the top of the image was oriented north. Two identical camera setups were used, and images were analyzed by two people. A common set of images were processed and analyzed to confirm a lack of camera or person bias. End of season destructive harvest was carried out on three representative center plants in each plot beginning 72 days after sowing. Plants were cut at the base, separated into leaf, stem, and reproductive tissues, and the following morphological traits were measured. Culm height was measured as the length from the base of the plant to the collar of the flag leaf on the first emerged tiller. Tiller height was measured as the length from the base of the plant to the collar of the flag leaf on the second emerged tiller. Basal circumference was measured with a length of twine wrapped around the root crown. Tiller number was measured as the count of tillers emerging from the bottommost node. Branch number was measured as the count of primary branches emerging from nodes one or higher. The separated leaf, stem, and reproductive tissues were dried at 65°C and weighed. Vegetative mass was calculated as the sum of leaf and stem mass. Total mass was calculated as the sum of leaf, stem, and panicle mass. All masses were standardized by planting density and are reported on a per unit ground area basis. The following R packages were used for data analysis and visualization: *ggplot2, plyr, reshape2,* and *ggrepel*. Data and scripts used in analyses are available in a.zip folder included in the Data S1. Raw hemispherical images are hosted on Dryad and are available for community use.

### 3.5 Data transformation

Data were normalized using a second power, square root, or cube root transformation. Normality was assessed through the R function *shapiro.test* and the associated histograms and Shapiro–Wilk's values. Results of the transformation procedures are shown in Table S1 **.**

### 3.6 Trait correlations

Trait correlations were tested using the R function *cor* using pairwise deletion to generate Pearson's coefficients of correlation and visualized with *corrplot*.

### 3.7 Principle component analysis

Principle component analysis was performed using the R function *prcomp,* with default parameters. A total of 176 genotypes—those with complete sets of observations—were used in the principle component analysis. Evaluation of individual eigenvectors for each trait was used to describe the significant PCs and used to parse the traits into biologically relevant groupings (Figure 2).

### 3.8 Quantitative trait loci analysis

Quantitative trait loci analysis was performed using “*foxy_qtl_pipeline,”* available at https://github.com/maxjfeldman/foxy_qtl_pipeline, written by Max Feldman and adapted from *R/qtl* (Broman, Wu, Sen, & Churchill, 2003; Feldman et al., 2017). QTL detection was performed using forward‐backward Haley–Knott regression in order to build a multiple QTL model from each trait. A genome scan interval of 1 cM and a window size of 10 were used. A total of 1,000 permutations were performed to estimate LOD threshold values. Additive effects were estimated as half the distance between phenotypic averages for the two homozygotes. To compare additive effects across traits with different scales, additive effects were normalized as a percent of the phenotypic mean (Des Marais, Hernandez, & Juenger, 2013). Colocalized QTL were grouped into “clusters” based on their mapping to same or neighboring markers where confidence intervals overlapped. Confidence intervals were calculated as the interval where the LOD score was within 1.5 units of its maximum. Lander and Botstein (1989) first proposed the use of two LOD support intervals and more recently Dupuis and Siegmund (1999) provided support for using the 1.5 LOD interval method (Dupuis & Siegmund, 1999; Lander & Botstein, 1989). The use of LOD supports intervals as a method to estimate the location of QTL and define colocalized clusters continues in current plant biology QTL experiments (Topp et al., 2013).

## AUTHOR CONTRIBUTIONS

A.D.B.L. conceived the original research plans. A.D.B.L., I.B., D.B., and R.E.P. supervised the experiments. H.J. provided seed for the mapping population. M.H. and H.S. collected validation data. D.B., R.E.P., and M.H. collected images and biomass data. D.B. and M.H. processed hemispherical images. R.E.P., M.F., and I.B. performed QTL analysis. D.B. and A.D.B.L. analyzed the data. D.B. and A.D.B.L. wrote the article; all authors reviewed and commented on the article.

## Supporting information


** **

** **

** **
