# Supplementary material for: High‐fidelity detection of crop biomass quantitative trait loci from low‐cost imaging in the field
Source: Plant Direct. 2018 Feb 22;2(2):e00041. doi: 10.1002/pld3.41 (PMC6508524; doi:10.1002/pld3.41)
Supplement: Supplementary file 1 [file PLD3-2-e00041-s001.pdf]

Table S1. Summary of phenotyping results. Mean, number of observations, minima, maxima, range, fold change, and summary of transformation procedures for 13 measured traits are reported.

Trait	Mean	N	Minimum	Maximum	Range	Fold change	Transformation	Wilks statistic	P-value
Branch number (count)	3.47	185	1.00	7.39	6.39	6.39	square root	0.99	0.1908
Clump spread (degrees)	3.93	203	2.99	4.89	1.90	0.64	cube root	0.99	0.6265
Culm height (mm)	20.20	188	10.25	27.47	17.22	1.68	square root	0.99	0.3258
Leaf mass (g)	9.07	189	4.41	16.69	12.28	2.79	square root	0.99	0.0459
PAI (m^2/m^2)	0.91	186	0.25	1.62	1.37	5.46	square root	0.98	0.0172
Panicle emergence (days after sowing)	39.17	210	29.00	49.00	20.00	0.69		0.93	0.0000
Panicle mass (g)	20.12	190	2.88	35.19	32.31	11.23	square root	0.99	0.4337
Reproductive to vegetative mass ratio	1.20	187	0.33	1.52	1.19	3.59	square root	0.93	0.0000
Stem mass (g)	14.22	191	6.87	22.49	15.62	2.27	square root	0.99	0.1684
Tiller height (mm)	359.68	187	113.67	721.67	608.00	5.35		0.99	0.1354
Tiller number (count)	1.81	187	1.26	2.69	1.43	1.14	cube root	0.99	0.0802
Total mass (g)	26.46	187	9.14	43.13	33.99	3.72	square root	0.99	0.5196
Vegetative mass (g)	16.92	188	8.44	28.01	19.57	2.32	square root	0.99	0.2685
