# Supplementary material for: High‐fidelity detection of crop biomass quantitative trait loci from low‐cost imaging in the field
Source: Plant Direct. 2018 Feb 22;2(2):e00041. doi: 10.1002/pld3.41 (PMC6508524; doi:10.1002/pld3.41)
Supplement: Supplementary file 2 [file PLD3-2-e00041-s002.pdf]

Table S2. Summary of QTL results. Marker name, location, proportion of genotypic variance explained, and relative additive effect for QTL identified for 13 measured traits at a 0.05 detection threshold are reported.

Trait	Marker	Chromosome	Position (cM)	LOD	Proportion of variance	Additive relative effect
Branch number	S1_36617026	1	84.01	6.18	8.9	-0.08
Branch number	S3_10844641	3	53.4	4.43	6.23	-0.07
Branch number	S5_7622386	5	44.09	5.38	7.67	-0.08
Branch number	S6_32411928	6	68.73	3.91	5.45	0.07
Branch number	S7_18218515	7	43.93	3.94	5.51	-0.07
Branch number	S9_6501061	9	33.94	3.74	5.21	-0.07
Branch number	S9_51926088	9	140.72	8.53	12.72	-0.1
Clump spread	S2_1368736	2	4.37	4.33	6.26	-0.03
Clump spread	S2_3532599	2	18	6.16	9.13	-0.04
Clump spread	S2_46536006	2	112.82	4.63	6.71	-0.02
Clump spread	S3_18761486	3	68.28	8.2	12.51	-0.03
Clump spread	S5_1722371	5	17.26	3.96	5.69	-0.02
Culm height	S2_37820883	2	70.03	7.21	13.12	0.06
Culm height	S5_42085155	5	100.66	3.99	6.92	-0.04
Culm height	S6_1367337	6	10.03	5.07	8.95	0.04
Culm height	S9_6295446	9	33.94	4.27	7.45	0.04
Culm height	S9_48859174	9	131.48	4.81	8.45	0.05
Leaf mass	S1_31298551	1	66.87	3.82	4.45	0.06
Leaf mass	S2_37965908	2	71.12	16.1	22.35	0.14
Leaf mass	S3_47360417	3	106.7	5.13	6.08	0.06
Leaf mass	S5_34517974	5	83.88	12.33	16.2	0.1
Leaf mass	S6_2365280	6	17.58	5.61	6.7	0.07
Leaf mass	S8_2959300	8	35.91	4.46	5.23	0.06
Leaf mass	S9_6724364	9	34.93	9.93	12.6	0.1
PAI	S2_37987785	2	71.12	10.94	18.47	0.13
PAI	S5_34517974	5	83.88	12.62	21.87	0.13
PAI	S6_2365280	6	17.58	4.73	7.27	0.08
PAI	S8_6974146	8	42.4	6.74	10.67	0.09

PAI	S9_6724364	9	34.93	4.27	6.52	0.08
Panicle emergence	S2_43829684	2	92.82	5.46	11.63	0.03
Panicle emergence	S7_32133319	7	99.94	5.13	10.87	0.04
Panicle mass	S2_41096419	2	80.96	5.79	12.09	0.1
Panicle mass	S5_34517974	5	83.88	5.22	10.83	0.09
Panicle mass	S7_32133319	7	99.94	6.27	13.19	-0.15
Reproductive to vegetative mass ratio	S5_4501200	5	34.5	5.22	8.49	0.05
Reproductive to vegetative mass ratio	S5_41999990	5	100.41	8.16	13.85	0.07
Reproductive to vegetative mass ratio	S6_7525462	6	40.63	7.76	13.08	-0.08
Reproductive to vegetative mass ratio	S7_32133319	7	99.94	7.18	12.01	-0.09
Second tiller height	S2_37820883	2	70.03	6.96	13.24	0.12
Second tiller height	S5_32800961	5	79.01	4.39	8.05	0.09
Second tiller height	S5_42113193	5	100.9	5.31	9.87	-0.1
Second tiller height	S6_1682903	6	12.47	5.59	10.43	0.09
Second tiller height	S9_6724364	9	34.93	4.46	8.19	0.09
Stem mass	S2_37820883	2	70.03	9.15	18.11	0.11
Stem mass	S5_29574617	5	69.56	4.39	8.12	0.06
Stem mass	S6_2516340	6	18.48	7.39	14.27	0.09
Total mass	S2_37820883	2	70.03	5.89	12.47	0.1
Total mass	S5_29574617	5	69.56	6.47	13.8	0.09
Total mass	S6_2516340	6	18.48	4.38	9.07	0.08
Vegetative mass	S2_37820883	2	70.03	11.56	22.02	0.12
Vegetative mass	S5_33682130	5	82.15	5.83	10.22	0.07
Vegetative mass	S6_2516340	6	18.48	7.77	14.01	0.09
Vegetative mass	S9_6724364	9	34.93	3.34	5.65	0.06
