# Supplementary material for: High‐fidelity detection of crop biomass quantitative trait loci from low‐cost imaging in the field
Source: Plant Direct. 2018 Feb 22;2(2):e00041. doi: 10.1002/pld3.41 (PMC6508524; doi:10.1002/pld3.41)
Supplement: Supplementary file 3 [file PLD3-2-e00041-s003.pdf]

Supplemental Information Guide

There are two supplemental tables:
Table S1. Summary of phenotyping results
Table S2. Summary of QTL results

Phenotype and QTL map visualization was performed using "PAI_analysis_availability.R" written by Darshi Banan.

QTL analysis was performed using "foxy_qtl_pipeline" written by Max Feldman, available at https://github.com/maxjfeldman/foxy_qtl_pipeline. An explanation of this pipeline is available at https://doi.org/10.1371/journal.pgen.1006841; Feldman MJ et al. 2017

Intermediate mappings results are contained in darshi_final_concatenated_summary_table.csv and are used to produce Table S2.

The following files contain phenotypic and genetic data:

1) "14LAI biomass.csv" – Raw data of above ground biomass used in validation experiment
   Column Definitions:
   - DATE: Sample collection date
   - RIL: Genotype identity
   - Sample: Tissue type
   - plot mass (g/m2): Dry tissue mass expressed on an meter square basis

2) "14LAI hemi.csv" – Raw data of hemispherical imaging used in validation experiment
   Column Definitions:
   - Date: Sample collection date
   - RIL: Genotype identity
   - PAI (m2/m2): Plant Area Index estimate from Hemiview

3) "14LAI imagej.csv" – Raw data of leaf area collected from ImageJ (NIH) used in validation experiment
   Column Definitions:
   - DATE: Sample collection date
   - RIL: Genotype identity
   - Total Area (cm2): Leaf area collected from a meter square ground

4) "pheno.hemi.csv" – Raw data of Plant Area Index used in mapping experiment.
   - plot: Plot identity within field
   - subplot_id: Subplot identity within plot
   - rep: Camera location either between "2" or "4" plants
   - data: Numeric value
   - trait: Plant Area Index (m^2/m^2)

5) "pheno_panicle.csv" – Raw data for panicle emergence used in mapping experiment.

- plot: Plot identity within field
- subplot_id: Subplot identity within plot
- data: Numeric value
- rep: Single observation
- trait: Panicle emergence Days After Sowing

6) “pheno_spread.csv” – Raw data for clump spread used in mapping experiment.
    - plot: Plot identity within field
    - subplot_id: Subplot identity within plot
    - data: Numeric value
    - rep: Subsampling 1-3
    - trait: Clump spread (degrees)

7) “pheno_traits.csv” – Raw data for harvest architectural traits used in mapping experiment
    - plot: Plot identity within field
    - subplot_id: Subplot identity within plot
    - rep: Subsampling 1-3
    - trait:
        i. Basal circumference (mm)
        ii. Branch number (count)
        iii. Culm height (mm)
        iv. Tiller1 height (mm; referred to as second tiller height in text)
        v. Tiller number (count)
    - data: Numeric value

8) “pheno_weights.csv” – Raw data
    - Plot: Plot identity within field
    - Subpot_id: Subplot identity within field
    - Trait:
        i. Leaf mass per plant (g)
        ii. Panicle mass per plant (g)
        iii. Stem mass per plant (g)
    - Data: Numeric value
    - Rep: Single observation

9) “GBS_map_A10xB100_v096.csv” – Genetic map used for QTL analysis and visualization
